# Novel Genetically Engineered Probiotics for Targeted Elimination of *Pseudomonas aeruginosa* in Intestinal Colonization

**DOI:** 10.3390/biomedicines11102645

**Published:** 2023-09-27

**Authors:** Hyun Kim, Ju Hye Jang, In Young Jung, Ha Rang Kim, Ju Hyun Cho

**Affiliations:** 1 Research Institute of Life Sciences, Gyeongsang National University, Jinju 52828, Republic of Korea; hyun.kim@gnu.ac.kr (H.K.); juhye.jang@gnu.ac.kr (J.H.J.); 2 Division of Applied Life Science (BK21Four), Gyeongsang National University, Jinju 52828, Republic of Korea; inyoung.jung@gnu.ac.kr (I.Y.J.); harang.kim@gnu.ac.kr (H.R.K.); 3 Division of Life Science, Gyeongsang National University, Jinju 52828, Republic of Korea

**Keywords:** antimicrobial resistance, antimicrobial peptide, *Pseudomonas aeruginosa*, engineered probiotics, *Escherichia coli* Nissle 1917

## Abstract

The intestinal carriage rates of *Pseudomonas aeruginosa* are notably elevated in immunosuppressed individuals and hospitalized patients, increasing the risk of infection and antibiotic-associated diarrhea. A potential solution to this issue lies in autonomous antibacterial therapy, remaining inactive until a pathogen is detected, and releasing antibacterial compounds on demand to eliminate the pathogen. This study focuses on the development of genetically engineered probiotics capable of detecting and eradicating *P. aeruginosa* by producing and secreting PA2-GNU7, a *P. aeruginosa*-selective antimicrobial peptide (AMP), triggered by the presence of *P. aeruginosa* quorum-sensing molecule *N*-(3-oxododecanoyl)-_L_-homoserine lactone (3OC_12_HSL). To achieve this goal, plasmid-based systems were constructed to produce AMPs in response to 3OC_12_HSL and secrete them into the extracellular medium using either the microcin V secretion system or YebF as a carrier protein. Following the transfer of these plasmid-based systems to *Escherichia coli* Nissle 1917 (EcN), we successfully demonstrated the ability of the engineered EcN to express and secrete PA2-GNU7, leading to the inhibition of *P. aeruginosa* growth in vitro. In addition, in a mouse model of intestinal *P. aeruginosa* colonization, the administration of engineered EcN resulted in reduced levels of *P. aeruginosa* in both the feces and the colon. These findings suggest that engineered EcN holds promise as a potential option for combating intestinal *P. aeruginosa* colonization, thus mitigating the risk of future endogenous infections in vulnerable patients.

## 1. Introduction

Antimicrobial resistance (AMR) has become a pressing global concern, posing a significant public threat in the 21st century. In 2019, bacterial AMR was responsible for 4.95 million deaths worldwide, with 1.27 million directly attributed to this phenomenon [1]. Unaddressed multidrug resistance (MDR) could result in 10 million annual global deaths by 2050 [2]. Among the most alarming MDR pathogens identified by the U.S. Centers for Disease Control and Prevention (CDC) and the World Health Organization (WHO), Gram-negative bacteria, including *Klebsiella pneumoniae*, *Acinetobacter baumannii*, and *Pseudomonas aeruginosa*, stand out as urgent unmet needs [3]. Of these pathogens, *P. aeruginosa*, a ubiquitous Gram-negative opportunistic pathogen, has emerged as a frequent cause of nosocomial infections, particularly in immunocompromised and critically ill patients [4,5]. While *P. aeruginosa* is not a typical member of the gut microbiome, the intestinal carriage rates of this bacterium significantly rise in immunosuppressed individuals after antibiotic treatment [6]. The intestinal colonization of *P. aeruginosa* is associated with an increased risk of developing *P. aeruginosa* infections. Notably, *P. aeruginosa* lung infections frequently occur in patients through the direct contamination of the lungs by gastrointestinal flora or via hematogenous spread from the intestine to the lungs [7,8,9,10]. Moreover, the intestinal translocation of *P. aeruginosa* can lead to antibiotic-associated diarrhea [11,12,13] and intestinal diseases accompanied by sepsis [14]. However, treating *P. aeruginosa* infections remains challenging due to its intrinsic resistance to many antibiotics and its ability to acquire resistance during therapy through various mechanisms [15]. Selectively eliminating *P. aeruginosa* from the gastrointestinal tract could hold the key to preventing lethal translocation events.

Antimicrobial peptides (AMPs) offer a promising alternative to traditional antibiotic molecules. Constituting a component of the innate immune system, AMPs are synthesized by plants, animals, and bacteria as the primary defense mechanism. Generally, AMPs are relatively short in length (less than 100 amino acids) and exhibit a cationic and amphiphilic nature, owing to the presence of lysine and arginine residues, as well as a high proportion (≥30%) of hydrophobic residues [16]. Unlike traditional antibiotics, these positively charged AMPs interact with the negatively charged bacterial cell membranes through electrostatic interactions, leading to membrane adsorption and conformational changes that result in bacterial death, reducing the likelihood of bacterial drug resistance [17]. Additionally, AMPs demonstrate rapid germ-killing abilities and low bactericidal concentrations, making them effective even against traditional antibiotic-resistant strains [18]. Despite these promising characteristics, the widespread clinical use of AMPs remains limited due to concerns about low stability, potential toxicity, and high production costs [19]. The oral delivery of AMPs to the gut poses particular challenges, as these molecules are susceptible to degradation before reaching the site of infection in sufficient quantities, leading to treatment failure [20].

Probiotics, such as *Escherichia coli* Nissle 1917 (EcN), are non-pathogenic microorganisms that can survive and thrive in the gastric environment, benefiting host health [21]. In recent years, engineering probiotics for the localized production and delivery of therapeutics in response to external signals has garnered considerable interest [22,23,24]. This approach offers substantial potential by enabling the efficient delivery of AMPs to the gut while bypassing degradation, addressing potential off-target effects, and significantly reducing the production costs of AMPs [23]. Various methods have been employed to produce and deliver AMPs to the gut from probiotics, including the use of fusion proteins coupled with secretion sequences [25,26,27,28,29,30] and inducing the lysis of the probiotic chassis to enable the release of peptides from within the bacteria [31,32].

Building on these advancements, we developed engineered strains of EcN capable of producing *P. aeruginosa*-selective AMPs in response to the *P. aeruginosa* quorum-sensing molecule *N*-(3-oxododecanoyl)-_L_-homoserine lactone (3OC_12_HSL). Our previous work yielded a novel α–helical membrane-active AMP named GNU7 (RLLRPLLQLLKQKLR) with enhanced stability and microbial cell specificity [33]. By adding a *P. aeruginosa*-targeting peptide (PA2) to GNU7, we achieved selectivity for *P. aeruginosa*, resulting in a hybrid peptide (PA2-GNU7) that demonstrated high specificity for *P. aeruginosa* while preferentially killing this pathogen over benign microorganisms [34]. Herein, we present plasmid-based systems designed to produce PA2-GNU7 in response to 3OC_12_HSL and secrete it into the extracellular medium using either the microcin V secretion system or YebF as a carrier protein. We transferred these plasmid-based systems to EcN and successfully demonstrated the engineered EcN’s ability to express and secrete PA2-GNU7, leading to the inhibition of *P. aeruginosa* growth.

## 2. Materials and Methods

### 2.1. Bacterial Strains and Culture

The bacterial strains and plasmids used in this study are listed in Table 1. *E. coli* TOP10 (Invitrogen, Carlsbad, CA, USA) was employed for cloning and protein expression. *P. aeruginosa* H103 (PAO1 wild-type prototroph) was generously provided by R. E. W. Hancock (University of British Columbia, Vancouver, BC, Canada). Clinically isolated *E. coli* KBN 12P06081 and carbapenemase-producing *P. aeruginosa* NCCP 14571 were obtained from the Gyeongsang National University Hospital Branch of the National Culture Collection for Pathogen (GNUH-NCCP) and the National Culture Collection for Pathogen (NCCP), respectively*. P. aeruginosa* H103 and NCCP 14571 are naturally resistant to 100 μg/mL of ampicillin. The probiotic *E. coli* Nissle 1917 (EcN) was acquired from Mutaflor (BL&H Co. Ltd., Seoul, Republic of Korea). Unless otherwise stated, commercial Luria-Bertani (LB) and yeast extract tryptone (YT) media were used for cloning and inhibition studies. Ampicillin (100 μg/mL) and kanamycin (50 μg/mL) were added to the culture media for antibiotic selection where required. All restriction and ligation enzymes were purchased from New England Biolabs (Ipswich, MA, USA) and Promega Corporation (Madison, WI, USA), respectively.

### 2.2. Plasmid Construction

Constitutive promoters (BBa_J23100, BBa_J23105, and BBa_J23118), along with the ribosome binding site (RBS) from pBb backbone (TTTAAGAAGGAGATATACAT), the *P. aeruginosa* transcription factor (*lasR*), the terminator (BBa_B0015), and the *lasI* promoter (P*_lasI_*, BBa_K649000) genes were chosen as components for developing sensor modules to detect 3OC_12_HSL. The reporter protein for the sensor modules was the red fluorescence protein (mRFP1). For constructing AMP secretion modules, the microcin V signal peptide (*SP_mccV_*), the microcin V secretion machinery (*cvaA* and *cvaB*), and the secretory protein (*yebF*) genes were selected. The sequences of BBa_J23100, BBa_J23105, BBa_J23118, BBa_B0015, and BBa_K649000 were taken from the iGEM parts registry. The sequences of *lasR* (NC_002516), *yebF* (NC_000913.3), microcin V signal peptide (*SP_mccV_*), *cvaA*, and *cvaB* (KX496988) were obtained from GenBank. The genetic constructions developed in this study were assembled using BglBrick standard synthetic biology protocols, unless otherwise stated [35].

For constructing plasmids containing sensor modules, sensor gene fragments that included constitutive promoters, RBS, codon-optimized *lasR*, terminator, and P*_lasI_* were synthesized at Bionics (Seoul, Republic of Korea) and cloned into the pBbE0k plasmid using *EcoR*I and *Xho*I restriction enzyme sites. Subsequently, the *mRFP1* gene from the pBbE1a plasmid (Addgene, MA, USA) was placed downstream of the *lasI* promoter to verify the sensor system’s ability to detect 3OC_12_HSL, leading to the creation of plasmids S100-RFP, S105-RFP, and S118-RFP.

To develop plasmids containing AMP secretion modules, a gene encoding SP_mccV_-PA2-GNU7 with an RBS and C-terminal 6 × His-tag was synthesized by Bionics and cloned into pBbE1a using *EcoR*I and *Xho*I restriction enzyme sites, resulting in plasmid P. Subsequently, the *cvaAB* gene, comprising a 3.6 kb fragment containing the *cvaA* and *cvaB* genes along with ~150 base pairs up- and downstream of the genes, was amplified from *E. coli* KBN 12P06081 by PCR and inserted downstream of the *SP_mccV_-PA2-GNU7* fusion gene, resulting in plasmid PAB. For the YebF fusion protein-based AMP secretion module, genes encoding YebF and YebF-(G_4_S)_2_-PA2-GNU7, each with an RBS and C-terminal 6 × His-tag, were synthesized by Bionics. These genes contained restriction sites for *BamH*I (5′ end) and *Xho*I (3′ end). After digestion with *BamH*I/*Xho*I, *yebF* and *yebF-(G_4_S)_2_-PA2-GNU7* fusion genes were cloned into the *Bgl*II/*Xho*I-digested pBbE1a plasmid, resulting in plasmids Y and YP, respectively.

Finally, the integrated plasmid systems, S100-PAB and S100-YP, were generated by integrating the AMP secretion modules (*SP_mccV_-PA2-GNU7-cvaAB* from plasmid PAB or synthesized *yebF-(G_4_S)_2_-PA2-GNU7*) downstream of the sensor module (S100). Sequence-verified plasmids were transformed into TOP10 or EcN cells for use in in vitro experiments and mouse experiments. A comprehensive list of plasmids and strains used in this study can be found in Table 1, while the primers utilized are listed in Table S1. The genetic parts employed in this study are summarized in Table S2.

### 2.3. Characterization of 3OC_12_HSL Sensor Module

Single colonies of EcN harboring the constructs (plasmids S100-RFP, S105-RFP, or S118-RFP) were inoculated into LB. After overnight growth, the cultures were diluted into fresh LB to a low OD and allowed to incubate further until an OD_600_ of 0.5 was reached before adding 3OC_12_HSL. Cultures were then transferred into a transparent flat-bottom 96-well plate in triplicate aliquots of 100 μL for induction with 3OC_12_HSL. The plate was incubated at 37 °C with moderate shaking for 3 h. The red fluorescence (excitation: 585/20 nm and emission: 620/10 nm) was read using Synergy^TM^ HTX Multi-Mode Microplate Reader (Bio-Tek, Winooski, VT, USA), and the result was zeroed with LB to remove background fluorescence.

RFP production induced by 3OC_12_HSL natively produced from *P. aeruginosa* was measured with EcN harboring the S100-RFP plasmid, as described above. Briefly, the diluted *P. aeruginosa* culture was incubated for 18 h at 37 °C and filtered with a 0.22 μm filter (Hyundai Micro, Seoul, Republic of Korea). Sterile filtrates containing 3OC_12_HSL were mixed with EcN sensor strain culture to activate RFP production. The resultant mixtures were transferred into a 96-well plate in triplicate aliquots of 100 μL and incubated at 37 °C with moderate shaking for 3 h. The RFP fluorescence was detected using a microplate reader (Bio-Tek).

### 2.4. Characterization of AMP Secretion Module for the Inducible Production of PA2-GNU7

Overnight cultures of *E. coli* TOP10 harboring the constructs (plasmids Y, YP, P, or PAB) were diluted in LB and harvested at an OD_600_ of 0.5. The collected cultures were induced with 0.1 mM isopropyl β-D-1-thiogalactopyranoside (IPTG) and incubated for 6 h at 37 °C. Subsequently, the cells were pelleted by centrifuge at 5000× *g* for 10 min. The pelleted cells were lysed with SDS-PAGE sample buffer by boiling for 5 min. The supernatants containing secreted proteins were collected and filter-sterilized using a 0.22 μm filter. Samples were separated by SDS-PAGE using the PeptiGel^TM^ I Peptide PAGE Analysis Kit (ELPIS-BIOTECH, Daejeon, Republic of Korea), and the immunoblotting assay was conducted as previously described [36]. The expressed AMPs were detected by incubation with a 6 × His-tag monoclonal antibody (Invitrogen) and a goat anti-mouse IgG (H + L) HRP-conjugated antibody (GenDEPOT, Katy, TX, USA).

### 2.5. 3OC_12_HSL-Inducible PA2-GNU7 Secretion by Engineered EcN

Overnight cultures of EcN harboring the integrated constructs (plasmids S100-PAB or S100-YP) were diluted in LB and harvested at an OD_600_ of 0.5. The collected cultures were induced with 0.1 μM 3OC_12_HSL and incubated for 6 h at 37 °C. The expression and secretion of PA2-GNU7 were confirmed by SDS-PAGE and immunoblotting analysis, as described above (Section 2.4).

### 2.6. Supernatant Activity Test

The supernatants were prepared by inducing the engineered EcNs for 6 h with 0.1 μM 3OC_12_HSL at an OD_600_ of 0.5. No antibiotics were added to the culture for this study. Engineered EcN cultures were pelleted by centrifugation at 5000× *g* for 10 min. Supernatants were filtered using a 0.22 μm filter into a sterile tube and stored at −20 °C until use. For the activity assay, overnight cultures of *P. aeruginosa* H103 and NCCP 14571 were diluted into fresh YT media to give 10^6^ CFU/mL. 30 μL of *P. aeruginosa* cultures were then added to 270 μL of sterilized engineered EcN supernatants and incubated at 37 °C for 3 h. One hundred microliters of serially diluted cultures were plated onto LB agar. The plates were incubated at 37 °C for 24 h, and the number of viable *P. aeruginosa* cells was assessed by counting CFUs. The same procedures were repeated for EcN containing the sensor construct (EcN S100-RFP) as a negative control. The percentage survival of *P. aeruginosa* was calculated relative to EcN S100-RFP.

### 2.7. P. aeruginosa Growth-Inhibition in Co-Culture with Engineered EcN

Engineered EcN and *P. aeruginosa* strains were diluted in YT media at an OD_600_ of 0.2. No antibiotics were added to the culture for this study. The engineered EcN and *P. aeruginosa* cultures were then combined in a 1:1 ratio and incubated at 37 °C for 6 h. A serial dilution of the co-cultures was plated on LB agar containing ampicillin (100 μg/mL) to enumerate *P. aeruginosa*. The percentage survival of *P. aeruginosa* was estimated, as described above (Section 2.6).

### 2.8. Animal Studies

All animal experiments were approved by the Animal Ethical Committee of Gyeongsang National University (GNU-230113-M0020). The evaluation of engineered EcN was conducted in a *P. aeruginosa*-infected mouse model, following the methods described by Hwang et al. with slight modifications [31]. Female ICR mice (6–8 weeks old) were given sterile drinking water containing penicillin G (1500 U/mL) and streptomycin sulfate (2 mg/mL) for 4 days. After 1 day of rest, food was withdrawn overnight, and mice were orally inoculated with 10^10^ CFU of *P. aeruginosa* (resuspended in 20% sucrose). At day 7 after *P. aeruginosa* infection, mice were randomly divided into four groups (*n* = 4–5 per group) and mock-treated or treated with 10^10^ CFU of each engineered EcN (EcN S100-RFP, EcN S100-PAB, and EcN S100-YP). Stool samples were collected 2–6 days after engineered EcN administration, weighed, and homogenized in PBS by vortexing. Homogenized fecal samples were serially diluted and plated on LB agar supplemented with ampicillin (100 μg/mL). After 24 h of incubation at 37 °C, colonies were counted, and CFUs were determined per gram of stool.

At day 6 after engineered EcN administration, mice were sacrificed, and the colon was dissected. Colon tissues were weighed and homogenized in PBS. Homogenized tissues were serially diluted in PBS and plated on LB agar supplemented with ampicillin. After 24 h of incubation, colonies were counted, and CFUs were determined per gram of tissue.

### 2.9. Statistical Analysis

Results are presented as mean ± s.e.m. unless stated otherwise. The Mann–Whitney U-test was used for the statistical evaluation of two experimental groups. To compare more than two groups, one-way ANOVA followed by Bonferroni’s post hoc test was used. All statistical evaluations were performed using GraphPad Prism software 5.0 (GraphPad Software, San Diego, CA, USA) with * *p* < 0.05, ** *p* < 0.01, and *** *p* < 0.001.

## 3. Results

### 3.1. Plasmid Design and Construction

**Figure 1:**
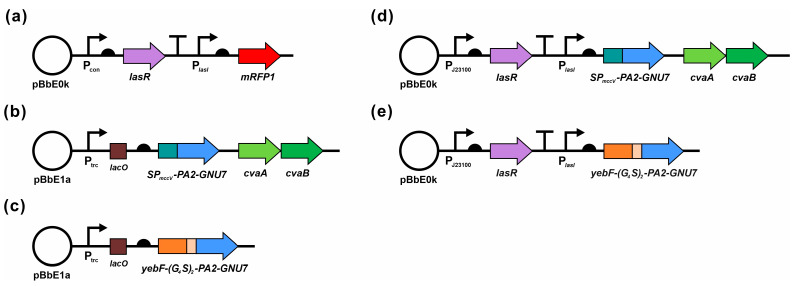
Schematic of plasmids used in this study. (**a**) Plasmid containing the sensor module (S100, S105, or S118) for 3OC_12_HSL recognition and downstream gene expression. mRFP1 was used as a reporter protein. (**b**,**c**) Plasmid PAB and YP for the IPTG-induced secretion of PA2-GNU7 and YebF-(G_4_S)_2_-PA2-GNU7, respectively. (**d**,**e**) Plasmid S100-PAB and S100-YP for the 3OC_12_HSL-induced secretion of PA2-GNU7 and YebF-(G_4_S)_2_-PA2-GNU7, respectively. P_con_: Constitutive promoter (J23100, J23105, or J23118); P*_lasI_*: *lasI* promoter; *lacO*: *lac* operator; *SP_mccV_*: microcin V signal peptide; *cvaA/B*: microcin V secretion machinery. The arrow size does not reflect the actual size of the genes.

The genetic framework of our plasmid-based system for engineering EcN consists of two interconnected modules: the sensor module and the AMP secretion module. The design of the sensor modules was rooted in the LasR-LasI quorum-sensing system of *P. aeruginosa*. These modules share a common gene circuit topology: a constitutive promoter drives the synthesis of a transcription factor LasR, while an inducible promoter (P*_lasI_*) facilitates the expression of downstream target genes upon activation by the LasR-3OC_12_HSL complex. We selected constitutive promoters J23100, J23105, and J23118, with varying predicted strengths (Figure 1a).

The AMP secretion modules were designed using two distinct export mechanisms: the micocin V secretion system and transport through a fusion partner. The microicin V secretion pathway involves the ATP-binding cassette transporter CvaB, the membrane fusion protein CvaA, and the outer membrane pore TolC [37]. For extracellular secretion, PA2-GNU7 was fused with an N-terminal microcin V signal peptide (SP_mccV_, MRTLTLNELDSVSGG), which is recognized and cleaved by the CvaB during transport to the extracellular milieu. Simultaneously, CvaAB secretion machinery is co-expressed (Figure 1b).

YebF, a 13 kDa protein with unspecified function encoded by the *yebGFE* operon, has been documented to be secreted into the extracellular medium by laboratory strains of *E. coli* after translocation into the periplasm and cleavage to a 10.8 kDa mature form by the sec-system [28,38]. In this study, we fused PA2-GNU7 with YebF via a flexible linker ((GGGGS) × 2) to ensure the extracellular secretion and proper functionality of PA2-GNU7 (Figure 1c).

Finally, complete systems were constructed by integrating plasmids containing the sensor and AMP secretion modules, which were then transferred to EcN. When exposed to *P. aeruginosa*, the sensor module recognizes 3OC_12_HSL secreted by *P. aeruginosa*, inducing downstream AMP secretion module expression. This leads to the secretion of PA2-GNU7 or YebF-(G_4_S)_2_-PA2-GNU7 into the extracellular medium (Figure 1d,e). The following sections detail the results obtained from characterizing individual modules, followed by demonstrating the anti-*Pseudomonas* activity exhibited by engineered EcN hosting the complete systems.

### 3.2. Characterization of the 3OC_12_HSL Sensor Module

**Figure 2:**
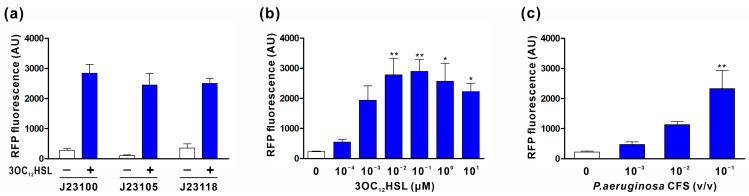
Characterization of the sensor module coupled with RFP reporter. (**a**) Comparative analysis of 3OC_12_HSL-mediated RFP expression using different constitutive promoters in the sensor module. EcN cells harboring the constructs (plasmids S100-RFP, S105-RFP, or S118-RFP) were induced with 1 μM 3OC_12_HSL for 3 h, and their corresponding fluorescence levels were quantified. (**b**) Quantification of RFP production at various 3OC_12_HSL concentrations in EcN cells harboring the S100-RFP plasmid. (**c**) Evaluation of RFP production in EcN cells harboring the S100-RFP plasmid in response to natively produced 3OC_12_HSL by *P. aeruginosa*. The data presented in all panels are depicted as mean ± s.e.m. from at least three independent experiments. Statistical significance was determined using one-way ANOVA followed by Bonferroni multiple comparison test, with significance levels denoted by asterisks (* *p* < 0.05 and ** *p* < 0.01).

We designed and tested a series of sensor plasmids in EcN to assess their ability to induce the expression of the mRFP1 reporter gene in response to exogenously supplied 3OC_12_HSL. All sensor modules recognized 3OC_12_HSL and led to mRFP1 expression. Notably, the sensor module containing P_J23100_ demonstrated higher RFP expression compared to those containing P_J23105_ and P_J23118_ (Figure 2a). As S100-RFP exhibited the most robust response to 3OC_12_HSL, we further evaluated the sensitivity of this sensor module to both exogenous and endogenous 3OC_12_HSL. EcN harboring the S100-RFP plasmid was incubated with varying concentrations of 3OC_12_HSL, and their fluorescence levels were measured. The results in Figure 2b indicate that 10 nM 3OC_12_HSL was sufficient to trigger an almost maximal response in EcN harboring the S100-RFP plasmid. Additionally, this cell responded to the cell-free culture supernatant (CFS) of *P. aeruginosa*, which contains naturally produced 3OC_12_HSL, suggesting that it has the capability of detecting *P. aeruginosa* cells effectively (Figure 2c). Thus, S100 was chosen as the sensor module for constructing engineered EcN.

### 3.3. Characterization of AMP Secretion Module for the Inducible Production of PA2-GNU7

**Figure 3:**
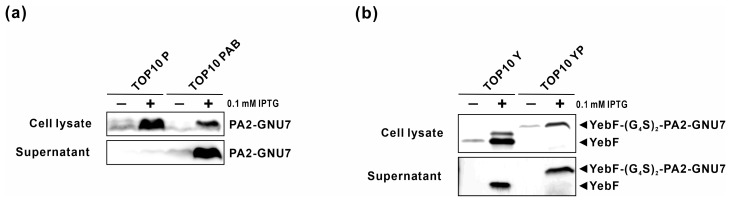
Analysis of intracellular and secreted AMPs from TOP10 cells harboring AMP secretion modules. Recombinant TOP10 cells were induced with IPTG for 6 h to assess the secretion of AMPs. The AMP secretion module was employed using either the microcin V secretion machinery (**a**) or YebF as a carrier protein (**b**). The TOP10 cells, designated as TOP10 P, TOP10 PAB, TOP10 Y, and TOP10 YP, carried plasmids P, PAB, Y, or YP, respectively. Confirmation of peptide secretion was achieved through Western blot analysis.

To enable the secretion of PA2-GNU7, we developed two distinct AMP secretion modules: one utilizing the microcin V secretion machinery (plasmid PAB) and the other using YebF as a carrier protein (plasmid YP). Before integrating these modules with the sensor module, we verified PA2-GNU7 production and secretion. The constructed modules (plasmids PAB and YP) were introduced into *E. coli* TOP10, and the cells were induced with IPTG. Western blotting was conducted to confirm PA2-GNU7 secretion. Plasmids containing only *SP_mccV_-PA2-GNU7* (plasmid P) or *yebF* (plasmid Y) were also examined for comparison. Figure 3a demonstrates the presence of PA2-GNU7 in both the cell lysate and the supernatant collected from TOP10 cells harboring the plasmid PAB (TOP10 PAB). However, TOP10 cells harboring the plasmid P (TOP10 P), which expresses only SP_mccV_-PA2-GNU7, were unable to secrete PA2-GNU7 into the extracellular medium. These findings confirm that the presence of PA2-GNU7 is due to secretion via the microcin V secretion machinery and not cell lysis. Similar observations were made with YebF-(G_4_S)_2_-PA2-GNU7 in the supernatant collected from TOP10 cells harboring the plasmid YP (TOP10 YP), underscoring the suitability of YebF as a carrier protein for the extracellular secretion of PA2-GNU7 (Figure 3b).

To verify the integrity of PA2-GNU7 in the supernatant, peptides from the supernatant of induced TOP10 PAB culture were purified by Ni-affinity chromatography and subjected to N-terminal sequencing (Figure S1). The N-terminal amino acid sequence of the purified peptide was determined through Edman degradation, identifying the first five amino acids as SQRKL, which correspond to the N-terminal sequence of PA2-GNU7. This confirms the proper cleavage of SP_mccV_ during secretion.

### 3.4. 3OC_12_HSL-Inducible PA2-GNU7 Secretion by Engineered EcN

**Figure 4:**
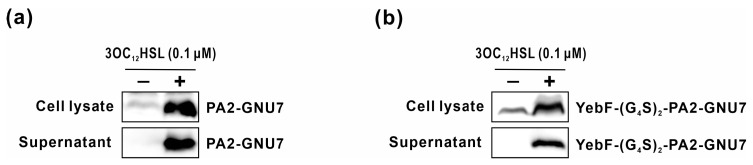
3OC_12_HSL-inducible AMP secretion by engineered EcN. Engineered EcNs cells harboring plasmids S100-PAB (**a**) or S100-YP (**b**) were induced with 3OC_12_HSL (0.1 μM) for 6 h. The secretion of PA2-GNU7 (**a**) or YebF-(G_4_S)_2_-PA2-GNU7 (**b**) into the extracellular medium was verified through Western blot analysis.

The complete systems (S100-PAB or S100-YP) were constructed by integrating the sensor and AMP secretion modules, enabling 3OC_12_HSL-dependent production and the secretion of PA2-GNU7. To validate successful 3OC_12_HSL-induced PA2-GNU7 secretion, engineered EcN harboring the complete system (S100-PAB or S100-YP) was induced with 3OC_12_HSL, and peptide secretion was confirmed via Western blotting. As depicted in Figure 4, PA2-GNU7 and YebF-(G_4_S)_2_-PA2-GNU7 were detected in the culture supernatant of the engineered EcN, affirming that these engineered cells produce and secrete AMPs in response to 3OC_12_HSL.

### 3.5. Verification of Engineered EcN Activity against P. aeruginosa

**Figure 5:**
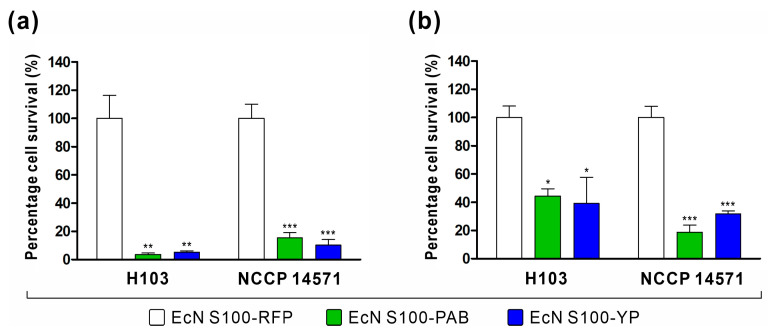
Analysis of the antimicrobial activity of engineered EcNs against *P. aeruginosa*. (**a**) Supernatant activity against *P. aeruginosa*. Exponential cultures of engineered EcN harboring plasmids S100-PAB or S100-YP were induced with 3OC_12_HSL (0.1 μM) for 6 h. Supernatants from these cultures were collected for antimicrobial activity testing. Cultures of *P. aeruginosa* were treated with sterile supernatants from the engineered EcN cells for 3 h, and the surviving *P. aeruginosa* cells were quantified. (**b**) Co-culture assay. Engineered EcN was co-cultured with *P. aeruginosa* cells at a 1:1 ratio for 6 h. Subsequently, the survival of *P. aeruginosa* cells was determined. The presented data illustrate the percentage of *P. aeruginosa* cell survival in each treatment group compared to the control group (EcN S100-RFP). The mean and s.e.m. are derived from three independent experiments. Statistical significance was determined using one-way ANOVA followed by Bonferroni multiple comparison test, with significance levels denoted by asterisks (* *p* < 0.05, ** *p* < 0.01, and *** *p* < 0.001).

To assess the anti-*Pseudomonas* activity of the engineered EcN cells, we conducted supernatant activity assays against *P. aeruginosa* strains H103 and antibiotic-resistant NCCP 14571. Supernatants from 3OC_12_HSL-induced engineered EcN cells harboring plasmids S100-PAB (EcN S100-PAB) or S100-YP (EcN S100-YP) were collected and applied to *P. aeruginosa* cultures. EcN harboring the sensor plasmid S100-RFP (EcN S100-RFP) was used as a control. The results in Figure 5a demonstrate that the supernatant from engineered EcNs exhibited significant activity against *P. aeruginosa*, including the antibiotic-resistant strain. Specifically, the supernatant from EcN S100-PAB (or EcN S100-YP) inhibited the growth of *P. aeruginosa* H103 and the antibiotic-resistant NCCP 14571 by 96.5% (94.8%) and 84.5% (89.8%), respectively. The supernatant from EcN harboring the plasmid S100-Y, which expresses YebF, did not have any effect against *P. aeruginosa* (data not shown).

The primary objective of this study was to engineer EcN to detect *P. aeruginosa* in the environment and subsequently secrete an AMP. To verify whether the engineered EcN strains can autonomously sense the presence of *P. aeruginosa* to initiate cell-killing, co-culture experiments were conducted with engineered EcNs and *P. aeruginosa*. The co-culture experiments were initiated with a ratio of approximately 1:1 between engineered EcNs (EcN S100-PAB or EcN S100-YP) and *P. aeruginosa* strains (H103 or NCCP 14571). Bacteria were grown for 6 h, and viable *P. aeruginosa* cells were measured. Both engineered EcNs demonstrated a remarkable inhibition of *P. aeruginosa* growth in this context. Specifically, EcN S100-PAB (or EcN S100-YP) inhibited the growth of *P. aeruginosa* H103 and the antibiotic-resistant NCCP 14571 by 55.7% (60.8%) and 81.3% (68.2%), respectively (Figure 5b). Taken together, engineered EcNs effectively responded to *P. aeruginosa* and inhibited its growth.

### 3.6. Evaluation of Engineered EcN in a P. aeruginosa-Infected Mouse Model

**Figure 6:**
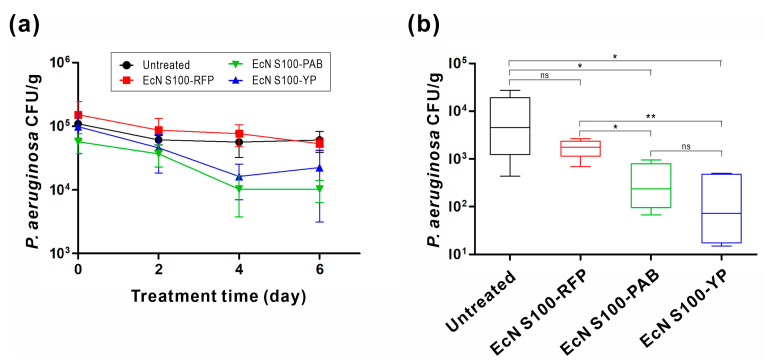
Evaluation of engineered EcNs in a mouse infection model. Mice subjected to *P. aeruginosa* infection were subsequently administered engineered EcNs. (**a**) Enumeration of *P. aeruginosa* cells in feces. *P. aeruginosa* cells in fecal samples were quantified for the duration of the experiment. (**b**) Total viable *P. aeruginosa* cells in colon samples at day 6 post-treatment. The data from two independent experiments are shown (*n* = 4–5). The data are presented in box-whisker plots, displaying the 90/10 percentile at the whiskers, the 75/25 percentile at the boxes, and the median at the centerline. Statistical significance was assessed using Mann–Whitney U-test, with significance levels denoted by asterisks (* *p* < 0.05 and ** *p* < 0.01). ”ns” indicates not significant (*p* > 0.05).

We further investigated the therapeutic potential of engineered EcNs in treating *P. aeruginosa* infections in the gastrointestinal tract. Engineered EcNs were administered to mice previously infected with *P. aeruginosa*, and the levels of *P. aeruginosa* in feces were monitored over time (Figure 6a). While the untreated and control group mice receiving EcN S100-RFP showed a gradual decline in fecal *P. aeruginosa* counts, the reduction in *P. aeruginosa* colonization was more pronounced in mice treated with engineered EcNs. After 4 days of treatment, the bacterial load was reduced by 82.1% for EcN S100-PAB and 83.5% for EcN S100-YP compared to the initial bacterial load before treatment. In contrast, the untreated and control groups exhibited only a 48.3% and 49.8% reduction in *P. aeruginosa* levels, respectively. At the end of the experiment, colon tissues were collected for analysis. The engineered EcN treatments, EcN S100-PAB and EcN S100-YP, resulted in a statistically significant reduction in bacterial load in the colon compared to the control group (Figure 6b).

## 4. Discussion

*P. aeruginosa* intestinal carriage rates are notably higher in immunosuppressed individuals and hospitalized patients, exposing them to an elevated risk of infections and antibiotic-associated diarrhea. The management of *P. aeruginosa* infections is further complicated by its multidrug resistance and antibiotic tolerance. As an alternative to conventional antibiotics, AMPs have emerged as promising options to combat antibiotic-resistant infections. Nonetheless, the practical application of AMPs faces obstacles, including limited stability and high production costs. Additionally, the potential for bacterial resistance is another major concern. Although AMPs are generally less likely to promote resistance, several mechanisms have been reported to contribute to resistance. These mechanisms encompass the expression of efflux pumps, surface modifications aimed at hindering electrostatic interactions between the membrane and peptides, and the increased secretion of proteolytic enzymes. Notably, prolonged exposure to a low concentration of AMP can induce resistance [39,40,41]. A potential solution lies in engineered probiotics equipped to detect external signals and subsequently produce and deliver AMPs only when and where needed.

Prior efforts have yielded various engineered microbes capable of detecting and eradicating *P. aeruginosa* through bacteriocin production and secretion. These engineered microbes utilize the *P. aeruginosa* transcription factor LasR as a sensor, which binds to quorum-sensing signals and triggers gene expression from its cognate promoter. Bacteriocins are delivered to the extracellular space by fusion proteins with secretion sequences or through the induced lysis of the probiotic chassis. For instance, Saeidi et al. engineered *E. coli* TOP10 to produce and release the bacteriocin pyocin S5 via cell lysis in response to *P. aeruginosa* quorum-sensing signals. They placed the E7 lysis protein, along with pyocin S5, under the control of P*_lux_*, enabling the efficient delivery of pyocin S5 [32]. Hwang et al. improved this system by adding the anti-biofilm enzyme dispersin B, also controlled by P*_las_* and implemented in EcN [31]. In another study, Hwang et al. engineered *E. coli* RP437 ∆*cheZ* to actively move towards and eliminate *P. aeruginosa*. They harnessed LasR-P*_las_* to regulate the expression of the chemotaxis protein CheZ, bacteriocin microcin S, and anti-biofilm enzyme DNaseI. They engineered microcin S and DNaseI to be secreted by YebF for extracellular delivery [42]. However, these bacteriocin-based systems are limited by potential resistance mechanisms and low specificity against *P. aeruginosa*. Notably, the effectiveness of S-type pyocins, which are narrow-spectrum bacteriocins produced by some *P. aeruginosa* strains, is hampered by the presence of cognate immunity proteins in the same strains [43,44]. Studies have shown that the presence of the pyocin S5-coding gene in about 24–25% of the clinical *P. aeruginosa* strains correlates with resistance to pyocin S5’s antimicrobial activity [45,46]. To address this, researchers have engineered probiotics to produce chimeric bacteriocins (pyocin–colicin) to circumvent recognition by immunity proteins [30,47]. Furthermore, while microcin S exhibits antimicrobial activity against *P. aeruginosa*, it might lead to dysbiosis in close relatives like *Enterobacteriaceae*, similar to other microcins produced by Gram-negative bacteria [48].

In this study, to overcome the limitations of bacteriocin-based systems, we employed PA2-GNU7, a hybrid antimicrobial peptide with high specificity for *P. aeruginosa* over benign microorganisms [34], as an anti-*Pseudomonas* agent in engineered EcN. In our engineered EcN, *P. aeruginosa* was detected by the sensor module using the transcription factor LasR and its cognate promoter P*_lasI_*. We optimized the module’s responsiveness by varying its constitutive promoter (Figure 1a and Figure 2a). The optimal sensor module (S100-RFP) effectively detected both exogenous and naturally produced 3OC_12_HSL from *P. aeruginosa*, exhibiting maximal response at 10 nM 3OC_12_HSL (Figure 2b,c). Considering that the accumulation of 3OC_12_HSL in the liquid culture of *P. aeruginosa* is estimated to exceed 1 uM [49,50], our sensor module holds the capability to detect *P. aeruginosa* cells effectively. Upon *P. aeruginosa* detection, PA2-GNU7 was produced and secreted using two different secretion modules: the microcin V secretion system or YebF as a carrier protein. The microcin V secretion system, a non-canonical type I secretion system, facilitated the direct secretion of PA2-GNU7 into the supernatant (Figure 3a, Figure 4a, and Figure S1). This system recognizes and cleaves a short N-terminal signal peptide SP_mccV_ during cargo export [51,52]. Several microcins from the microcin V secretion system have been successfully produced and secreted independently from each construct using SP_mccV_ [25,27]. The YebF protein, exported by the Sec pathway, aided the extracellular secretion of various proteins, including AMPs [30,53]. Herein, we fused PA2-GNU7 with YebF via a long flexible linker ((GGGGS) × 2) and verified the secretion of YebF-(G_4_S)_2_-PA2-GNU7 into the supernatant (Figure 3b and Figure 4b).

Integrating the sensor and AMP secretion modules into engineered EcN led to significant activity against *P. aeruginosa*, including antibiotic-resistant strains. The supernatant from 3OC_12_HSL-induced engineered EcN, harboring complete systems (S100-PAB or S100-YP), effectively hindered the growth of *P. aeruginosa* H103 and the antibiotic-resistant NCCP 14571 (Figure 5a). In addition, the co-culture inhibition assay, which emulates a more realistic environment in which engineered EcN and *P. aeruginosa* compete within the same culture, demonstrated the inhibition of *P. aeruginosa* growth, consistent with the supernatant assay results (Figure 5b). As mentioned above, EcN S100-PAB and EcN S100-YP secreted cargo using the microcin V secretion system or transported through the fusion partner YebF, respectively. Notably, the SP_mccV_ is not retained by the exported cargo in the microcin V secretion system. Consequently, EcN S100-PAB secretes PA2-GNU7 in its native form, ensuring the antimicrobial function of PA2-GNU7. On the other hand, EcN S100-YP secretes PA2-GNU7 as a YebF-(G_4_S)_2_-PA2-GNU7 fusion protein. Despite prior concerns that the addition of a secretion carrier protein to a bacteriocin might compromise its antimicrobial activity [30,42], our findings suggest that the fusion protein YebF-(G_4_S)_2_-PA2-GNU7 effectively retained potent anti-*Pseudomonas* activity. This could be attributed to the long flexible linker between YebF and PA2-GNU7, enabling interaction with *P. aeruginosa* cell membranes. Likewise, Sun et al. reported that the activity of fusion protein is dependent on a long flexible linker between the AMP and carrier protein. In this work, the long flexible linker between DAMP4 (carrier protein) and pexiganan (AMP) acted as a spacer, separating pexiganan and DAMP4, thereby facilitating the exposure of the pexiganan motif to target membranes for membrane disruption [54].

The in vivo therapeutic efficacy of our engineered EcN strains was assessed in a mouse model of *P. aeruginosa* colonization. As shown in Figure 6, the administration of EcN S100-PAB and EcN S100-YP led to diminished levels of *P. aeruginosa* in both feces and the colon. Although EcN naturally produces two bacteriocins, microcin H47 and microcin M, their antibacterial activity is confined to iron-limited mediums and specific species of *Enterobacteriaceae* [55,56,57]. Hence, the observed suppression of *P. aeruginosa* growth might be primarily attributed to the production of PA2-GNU7 (or YebF-(G_4_S)_2_-PA2-GNU7) by engineered EcN strains as opposed to the action of naturally produced microcins.

## 5. Conclusions

In summary, our study presents a novel approach, employing *P. aeruginosa* quorum-sensing molecule-responsive engineered EcNs to produce and deliver PA2-GNU7, a *P. aeruginosa*-selective AMP. These engineered EcN strains effectively produced PA2-GNU7 upon sensing 3OC_12_-HSL and secreted it extracellularly through either the microcin V secretion system or YebF as a carrier protein, leading to notable inhibition of *P. aeruginosa* growth in vitro. Furthermore, these engineered EcN strains displayed promising potential in reducing intestinal *P. aeruginosa* colonization in a mouse model. Though further optimization is required, our engineered EcN holds significant promise as a potential clinical treatment for *P. aeruginosa* infections.

## Figures and Tables

**Table 1:** Bacterial strains and plasmids used in this study.

Strain/Plasmid	Description	Source
*E. coli* TOP10	Host strain used for cloning and AMP expression	Invitrogen
*E. coli* Nissle 1917	Nonpathogenic human commensal used in probiotics	Mutaflor
*P. aeruginosa* H103	PAO1 wild-type prototroph	University of British Columbia
*P. aeruginosa* NCCP 14571	Meropenem^R^, ceftazidime^R^, tobramycin^R^, gentamicin^R^, amikacin^R^, cefepime^R^, cefotaxime^R^, ciprofloxacin^R^, imipenem^R^, piperacillin^R^, piperacillin-tazobactam^R^	NCCP
pBbE0k-RFP	Kan^R^, ColE1, constitutive mRFP1	Addgene
pBbE1a-RFP	Amp^R^, ColE1, constitutive LacI (*lacIq*), P_trc_-controlled mRFP1	Addgene
S100-RFP	pBbE0k, constitutive LasR (J23100), P*_lasI_*-controlled mRFP1	This study
S105-RFP	pBbE0k, constitutive LasR (J23105), P*_lasI_*-controlled mRFP1	This study
S118-RFP	pBbE0k, constitutive LasR (J23118), P*_lasI_*-controlled mRFP1	This study
Y	pBbE1a, P_trc_-controlled YebF with C-terminal 6 × His-tag	This study
YP	pBbE1a, P_trc_-controlled YebF-(G_4_S)_2_-PA2-GNU7 fusion protein with C-terminal 6 × His-tag	This study
P	pBbE1a, P_trc_-controlled PA2-GNU7 with N-terminal microcin V signal peptide (SP_mccV_) and C-terminal 6 × His-tag	This study
PAB	pBbE1a, P_trc_-controlled SP_mccV_-PA2-GNU7 with C-terminal 6 × His-tag and CvaA/B	This study
S100-YP	pBbE0k, constitutive LasR (J23100), P*_lasI_*-controlled YebF-(G_4_S)_2_-PA2-GNU7 fusion protein with C-terminal 6 × His-tag	This study
S100-PAB	pBbE0k, constitutive LasR (J23100), P*_lasI_*-controlled SP_mccV_-PA2-GNU7 with C-terminal 6 × His-tag and CvaA/B	This study

## Data Availability

Not applicable.

## Supplementary Materials

The following supporting information can be downloaded at: https://www.mdpi.com/article/10.3390/biomedicines11102645/s1, Figure S1: Purification of PA2-GNU7 from the culture supernatant of TOP10 harboring the plasmid PAB and determination of N-terminal amino acid sequences; Table S1: List of oligonucleotides used in this study; Table S2: DNA sequences of genes used in this study.

## References

[1] Antimicrobial Resistance Collaborators (2022). Global burden of bacterial antimicrobial resistance in 2019: A systematic analysis. Lancet.

[2] O’Neil J. (2016). Trackling Drug-Resistant Infections Globally: Final Report and Recommendations. London: Review on Antimicrobial Resistance. https://amr-review.org/Publications.html.

[3] Prasad N.K., Seiple I.B., Cirz R.T., Rosenberg O.S. (2022). Leaks in the Pipeline: A Failure Analysis of Gram-Negative Antibiotic Development from 2010 to 2020. Antimicrob. Agents Chemother..

[4] Kang C.I., Kim S.H., Kim H.B., Park S.W., Choe Y.J., Oh M.D., Kim E.C., Choe K.W. (2003). *Pseudomonas aeruginosa* bacteremia: Risk factors for mortality and influence of delayed receipt of effective antimicrobial therapy on clinical outcome. Clin. Infect. Dis..

[5] De Bentzmann S., Plesiat P. (2011). The *Pseudomonas aeruginosa* opportunistic pathogen and human infections. Environ. Microbiol..

[6] Janapatla R.P., Dudek A., Chen C.L., Chuang C.H., Chien K.Y., Feng Y., Yeh Y.M., Wang Y.H., Chang H.J., Lee Y.C. (2023). Marine prebiotics mediate decolonization of *Pseudomonas aeruginosa* from gut by inhibiting secreted virulence factor interactions with mucins and enriching Bacteroides population. J. Biomed. Sci..

[7] Gomez-Zorrilla S., Camoez M., Tubau F., Canizares R., Periche E., Dominguez M.A., Ariza J., Pena C. (2015). Prospective observational study of prior rectal colonization status as a predictor for subsequent development of *Pseudomonas aeruginosa* clinical infections. Antimicrob. Agents Chemother..

[8] Wheatley R.M., Caballero J.D., van der Schalk T.E., De Winter F.H.R., Shaw L.P., Kapel N., Recanatini C., Timbermont L., Kluytmans J., Esser M. (2022). Gut to lung translocation and antibiotic mediated selection shape the dynamics of *Pseudomonas aeruginosa* in an ICU patient. Nat. Commun..

[9] Markou P., Apidianakis Y. (2014). Pathogenesis of intestinal *Pseudomonas aeruginosa* infection in patients with cancer. Front. Cell Infect. Microbiol..

[10] Okuda J., Hayashi N., Okamoto M., Sawada S., Minagawa S., Yano Y., Gotoh N. (2010). Translocation of *Pseudomonas aeruginosa* from the intestinal tract is mediated by the binding of ExoS to an Na,K-ATPase regulator, FXYD3. Infect. Immun..

[11] Kim S.W., Peck K.R., Jung S.I., Kim Y.S., Kim S., Lee N.Y., Song J.H. (2001). *Pseudomonas aeruginosa* as a potential cause of antibiotic-associated diarrhea. J. Korean Med. Sci..

[12] Hoff R.T., Patel A., Shapiro A. (2020). *Pseudomonas aeruginosa*: An Uncommon Cause of Antibiotic-Associated Diarrhea in an Immunocompetent Ambulatory Adult. Case Rep. Gastrointest. Med..

[13] Chuang C.H., Janapatla R.P., Wang Y.H., Chang H.J., Huang Y.C., Lin T.Y., Chiu C.H. (2017). *Pseudomonas aeruginosa*-associated Diarrheal Diseases in Children. Pediatr. Infect. Dis. J..

[14] Chuang C.H., Wang Y.H., Chang H.J., Chen H.L., Huang Y.C., Lin T.Y., Ozer E.A., Allen J.P., Hauser A.R., Chiu C.H. (2014). Shanghai fever: A distinct *Pseudomonas aeruginosa* enteric disease. Gut.

[15] Ibrahim D., Jabbour J.F., Kanj S.S. (2020). Current choices of antibiotic treatment for *Pseudomonas aeruginosa* infections. Curr. Opin. Infect. Dis..

[16] Hancock R.E., Sahl H.G. (2006). Antimicrobial and host-defense peptides as new anti-infective therapeutic strategies. Nat. Biotechnol..

[17] Mookherjee N., Hancock R.E. (2007). Cationic host defence peptides: Innate immune regulatory peptides as a novel approach for treating infections. Cell. Mol. Life Sci..

[18] Lei J., Sun L., Huang S., Zhu C., Li P., He J., Mackey V., Coy D.H., He Q. (2019). The antimicrobial peptides and their potential clinical applications. Am. J. Transl. Res..

[19] Roca-Pinilla R., Lisowski L., Aris A., Garcia-Fruitos E. (2022). The future of recombinant host defense peptides. Microb. Cell Factories.

[20] Gardiner G.E., Rea M.C., O’Riordan B., O’Connor P., Morgan S.M., Lawlor P.G., Lynch P.B., Cronin M., Ross R.P., Hill C. (2007). Fate of the two-component lantibiotic lacticin 3147 in the gastrointestinal tract. Appl. Environ. Microbiol..

[21] Gareau M.G., Sherman P.M., Walker W.A. (2010). Probiotics and the gut microbiota in intestinal health and disease. Nat. Rev. Gastroenterol. Hepatol..

[22] Zhou Z., Chen X., Sheng H., Shen X., Sun X., Yan Y., Wang J., Yuan Q. (2020). Engineering probiotics as living diagnostics and therapeutics for improving human health. Microb. Cell Factories.

[23] Mejia-Pitta A., Broset E., de la Fuente-Nunez C. (2021). Probiotic engineering strategies for the heterologous production of antimicrobial peptides. Adv. Drug Deliv. Rev..

[24] Pedrolli D.B., Ribeiro N.V., Squizato P.N., de Jesus V.N., Cozetto D.A., Tuma R.B., Gracindo A., Cesar M.B., Freire P.J., da Costa A.F. (2019). Engineering Microbial Living Therapeutics: The Synthetic Biology Toolbox. Trends Biotechnol..

[25] Geldart K., Forkus B., McChesney E., McCue M., Kaznessis Y.N. (2016). pMPES: A Modular Peptide Expression System for the Delivery of Antimicrobial Peptides to the Site of Gastrointestinal Infections Using Probiotics. Pharmaceuticals.

[26] Volzing K., Borrero J., Sadowsky M.J., Kaznessis Y.N. (2013). Antimicrobial peptides targeting Gram-negative pathogens, produced and delivered by lactic acid bacteria. ACS Synth. Biol..

[27] Geldart K.G., Kommineni S., Forbes M., Hayward M., Dunny G.M., Salzman N.H., Kaznessis Y.N. (2018). Engineered *E. coli* Nissle 1917 for the reduction of vancomycin-resistant *Enterococcus* in the intestinal tract. Bioeng. Transl. Med..

[28] Zhang G., Brokx S., Weiner J.H. (2006). Extracellular accumulation of recombinant proteins fused to the carrier protein YebF in *Escherichia coli*. Nat. Biotechnol..

[29] Borrero J., Chen Y., Dunny G.M., Kaznessis Y.N. (2015). Modified lactic acid bacteria detect and inhibit multiresistant Enterococci. ACS Synth. Biol..

[30] Gupta S., Bram E.E., Weiss R. (2013). Genetically programmable pathogen sense and destroy. ACS Synth. Biol..

[31] Hwang I.Y., Koh E., Wong A., March J.C., Bentley W.E., Lee Y.S., Chang M.W. (2017). Engineered probiotic *Escherichia* coli can eliminate and prevent *Pseudomonas aeruginosa* gut infection in animal models. Nat. Commun..

[32] Saeidi N., Wong C.K., Lo T.M., Nguyen H.X., Ling H., Leong S.S., Poh C.L., Chang M.W. (2011). Engineering microbes to sense and eradicate *Pseudomonas aeruginosa*, a human pathogen. Mol. Syst. Biol..

[33] Kim H., Jang J.H., Kim S.C., Cho J.H. (2014). De novo generation of short antimicrobial peptides with enhanced stability and cell specificity. J. Antimicrob. Chemother..

[34] Kim H., Jang J.H., Kim S.C., Cho J.H. (2020). Development of a novel hybrid antimicrobial peptide for targeted killing of *Pseudomonas aeruginosa*. Eur. J. Med. Chem..

[35] Lee T.S., Krupa R.A., Zhang F., Hajimorad M., Holtz W.J., Prasad N., Lee S.K., Keasling J.D. (2011). BglBrick vectors and datasheets: A synthetic biology platform for gene expression. J. Biol. Eng..

[36] Jang J.H., Kim H., Jung I.Y., Cho J.H. (2021). A20 Inhibits LPS-Induced Inflammation by Regulating TRAF6 Polyubiquitination in Rainbow Trout. Int. J. Mol. Sci..

[37] Smith T.J., Sondermann H., O’Toole G.A. (2018). Type 1 Does the Two-Step: Type 1 Secretion Substrates with a Functional Periplasmic Intermediate. J. Bacteriol..

[38] Burdette L.A., Leach S.A., Wong H.T., Tullman-Ercek D. (2018). Developing Gram-negative bacteria for the secretion of heterologous proteins. Microb. Cell Factories.

[39] Andersson D.I., Hughes D., Kubicek-Sutherland J.Z. (2016). Mechanisms and consequences of bacterial resistance to antimicrobial peptides. Drug Resist. Updat..

[40] Mwangi J., Hao X., Lai R., Zhang Z.Y. (2019). Antimicrobial peptides: New hope in the war against multidrug resistance. Zool. Res..

[41] Luong H.X., Ngan H.D., Thi Phuong H.B., Quoc T.N., Tung T.T. (2022). Multiple roles of ribosomal antimicrobial peptides in tackling global antimicrobial resistance. R. Soc. Open Sci..

[42] Hwang I.Y., Tan M.H., Koh E., Ho C.L., Poh C.L., Chang M.W. (2014). Reprogramming microbes to be pathogen-seeking killers. ACS Synth. Biol..

[43] Rasouliha B.H., Ling H., Ho C.L., Chang M.W. (2013). A predicted immunity protein confers resistance to pyocin S5 in a sensitive strain of *Pseudomonas aeruginosa*. ChemBioChem.

[44] Paskevicius S., Starkevic U., Misiunas A., Vitkauskiene A., Gleba Y., Razanskiene A. (2017). Plant-expressed pyocins for control of *Pseudomonas aeruginosa*. PLoS ONE.

[45] Paskevicius S., Dapkute V., Misiunas A., Balzaris M., Thommes P., Sattar A., Gleba Y., Razanskiene A. (2022). Chimeric bacteriocin S5-PmnH engineered by domain swapping efficiently controls *Pseudomonas aeruginosa* infection in murine keratitis and lung models. Sci. Rep..

[46] Snopkova K., Dufkova K., Klimesova P., Vanerkova M., Ruzicka F., Hola V. (2020). Prevalence of bacteriocins and their co-association with virulence factors within *Pseudomonas aeruginosa* catheter isolates. Int. J. Med. Microbiol..

[47] Gao Y., Wei J., Pu L., Fu S., Xing X., Zhang R., Jin F. (2023). Remotely Controllable Engineered Bacteria for Targeted Therapy of *Pseudomonas aeruginosa* Infection. ACS Synth. Biol..

[48] Pons A.M., Delalande F., Duarte M., Benoit S., Lanneluc I., Sable S., Van Dorsselaer A., Cottenceau G. (2004). Genetic analysis and complete primary structure of microcin L. Antimicrob. Agents Chemother..

[49] Scholz R.L., Greenberg E.P. (2017). Positive Autoregulation of an Acyl-Homoserine Lactone Quorum-Sensing Circuit Synchronizes the Population Response. mBio.

[50] Huang J.J., Han J.I., Zhang L.H., Leadbetter J.R. (2003). Utilization of acyl-homoserine lactone quorum signals for growth by a soil pseudomonad and *Pseudomonas aeruginosa* PAO1. Appl. Environ. Microbiol..

[51] Kim S.Y., Parker J.K., Gonzalez-Magaldi M., Telford M.S., Leahy D.J., Davies B.W. (2023). Export of Diverse and Bioactive Small Proteins through a Type I Secretion System. Appl. Environ. Microbiol..

[52] Parker J.K., Davies B.W. (2022). Microcins reveal natural mechanisms of bacterial manipulation to inform therapeutic development. Microbiology.

[53] Seo E.J., Weibel S., Wehkamp J., Oelschlaeger T.A. (2012). Construction of recombinant *E. coli* Nissle 1917 (EcN) strains for the expression and secretion of defensins. Int. J. Med. Microbiol..

[54] Sun B., Wibowo D., Sainsbury F., Zhao C.X. (2018). Design and production of a novel antimicrobial fusion protein in *Escherichia coli*. Appl. Microbiol. Biotechnol..

[55] Mortzfeld B.M., Palmer J.D., Bhattarai S.K., Dupre H.L., Mercado-Lubio R., Silby M.W., Bang C., McCormick B.A., Bucci V. (2022). Microcin MccI47 selectively inhibits enteric bacteria and reduces carbapenem-resistant *Klebsiella pneumoniae* colonization in vivo when administered via an engineered live biotherapeutic. Gut Microbes.

[56] Vassiliadis G., Destoumieux-Garzon D., Lombard C., Rebuffat S., Peduzzi J. (2010). Isolation and characterization of two members of the siderophore-microcin family, microcins M and H47. Antimicrob. Agents Chemother..

[57] Sassone-Corsi M., Nuccio S.P., Liu H., Hernandez D., Vu C.T., Takahashi A.A., Edwards R.A., Raffatellu M. (2016). Microcins mediate competition among Enterobacteriaceae in the inflamed gut. Nature.

