# Supplementary material for: Novel Genetically Engineered Probiotics for Targeted Elimination of Pseudomonas aeruginosa in Intestinal Colonization
Source: Biomedicines. 2023 Sep 27;11(10):2645. doi: 10.3390/biomedicines11102645 (PMC10604247; doi:10.3390/biomedicines11102645)
Supplement: Supplementary file 1 [file biomedicines-11-02645-s001.zip › biomedicines-2608585-supplementary.pdf]

biomedicines

Supplementary Information

Novel Genetically Engineered Probiotics for Targeted Elimination of *Pseudomonas aeruginosa* in Intestinal Colonization

Hyun Kim [1], Ju Hye Jang [1], In Young Jung [2], Ha Rang Kim [2] and Ju Hyun Cho [1,2,3,*]

1 Research Institute of Life Sciences, Gyeongsang National University, Jinju 52828, Republic of Korea; hyun.kim@gnu.ac.kr (H.K.); juhye.jang@gnu.ac.kr (J.H.J.)
2 Division of Applied Life Science (BK21Four), Gyeongsang National University, Jinju 52828, Republic of Korea; inyoung.jung@gnu.ac.kr (I.Y.J.); harang.kim@gnu.ac.kr (H.R.K.)
3 Division of Life Science, Gyeongsang National University, Jinju 52828, Republic of Korea
* Correspondence: juhyun.cho@gnu.ac.kr; Tel.: +82-55-772-1347; Fax: +82-55-772-1349

Supplementary method

Purification of Secreted AMP and N-terminal Sequencing

To purify AMPs, a 500 mL culture of TOP10 cells harboring the plasmid PAB (TOP10 PAB) was grown until reaching an OD_{600} of 0.5. AMP expression was induced by treating the culture with 1 mM IPTG for 24 h at 37 °C. Subsequently, the culture supernatant was harvested via centrifugation at 5000× *g* 10 min and sterile filtered (0.22-µm, Hyundai Micro, Seoul, Republic of Korea). Peptides from the culture supernatant were precipitated with 80% ammonium sulfate and pelleted through centrifugation at 10000× *g* for 10 min. The resultant pellet was resuspended and dialyzed in a binding buffer containing 20 mM sodium phosphate (pH 7.4), 300 mM NaCl, 10 mM imidazole, and 6 M Urea. Purification was then conducted using affinity chromatography with His-bind nickel resin (Thermo Fisher Scientific, Waltham, MA, USA) according to the manufacturer's instructions. The efficacy of PA2-GNU7 purification was confirmed through SDS-PAGE analysis and Coomassie blue staining.

After purification, the PA2-GNU7 peptide was resolved on a 15% SDS-PAGE gel and transferred onto a polyvinylidene difluoride (PVDF) membrane. The protein transfer was confirmed by Coomassie blue staining, and the band corresponding to purified PA2-GNU7 was subjected to N-terminal sequencing via Edman degradation, performed by the Life Science Laboratory Corporation (Seoul, Republic of Korea).

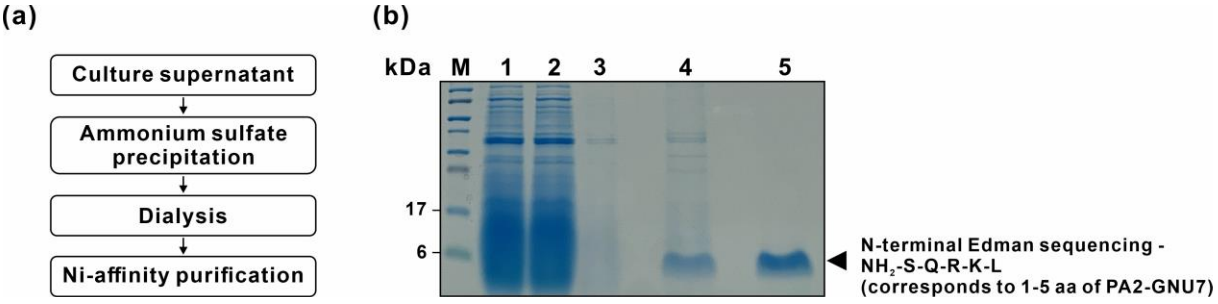

Figure S1. Purification of PA2-GNU7 from the culture supernatant of TOP10 harboring the plasmid PAB and determination of N-terminal amino acid sequences. (a) Schematic diagram of the purification process. (b) SDS-PAGE analysis of purified PA2-GNU7. The culture supernatant of IPTG-induced TOP10 PAB was analyzed after dialysis (lane 1) and affinity purification (lane 2-5). M: molecular weight marker; 2: Flowthrough fraction; 3: Wash fraction; 4: First elution fraction; 5: Second elution fraction. The purified peptide was transferred onto a PVDF membrane, and the N-terminal amino acid sequence was determined through Edman degradation, identifying the first five amino acids as SQRKL, which correspond to the N-terminal sequence of PA2-GNU7.

Table S1. List of oligonucleotides used in this study

Primer	Sequence (5′-3′)
CvaAB-FWD	GAATTCAAAAGATCTTCTCCTATGTTGTATGTTTATATGATTTTCCTTGAAAC
CvaAB-REV	CTCGAGTTTGGATCCGAAAGAACAGTTATTGGACAATCCACACAG

Table S2. DNA sequences of genes used in this study.

Gene	Sequence (5′-3′)*
Codon-optimized *lasR*	**tttaagaaggagatatacat**ATGGCTCTAGTAGATGGATTTTTGGAATTAGAGCGCTCTAGCGGTAAG<br>CTGGAATGGAGCGCTATTTTGCAAAAAATGGCGAGCGACCTGGGCTTTAGCAAGATCCTGT<br>TTGGCTTGCTGCCGAAAGATTCGCAAGATTACGAGAACGCGTTCATTGTTGGTAATTACCC<br>GGCAGCTTGGAGAGAACATTATGACCGCGCAGGCTACGCCCGTGTGGACCCGACCGTTTC<br>CCACTGCACCCAGTCCGTGCTGCCGATTTTCTGGGAACCGAGCATTTATCAGACCCGTAAG<br>CAGCATGAATTTTTCGAGGAAGCGTCGGCTGCCGGTTTGGTTTATGGTCTGACCATGCCGCT<br>GCATGGCGCGCGTGGTGAACTGGGTGCGCTCTCCCTGAGCGTGGAAGCGGAAAATCGTGC<br>GGAGGCGAACCGTTTTATGGAAAGCGTGCTCCCAACGCTGTGGATGCTGAAAGACTACGC<br>TCTGCAGTCAGGCGCGGGCTTAGCATTCGAGCACCCGGTGAGCAAACCGGTCGTGTTGAC<br>GTCTCGCGAGAAAGAGGTTCTGCAATGGTGTGCAATCGGTAAGACTTCCTGGGAGATCAG<br>CGTCATCTGCAACTGCAGCGAGGCTAATGTTAACTTCCACATGGGTAACATTCGTCGTAAG<br>TTCGGCGTTACCAGTCGTCGCGTAGCGGCGATCATGGCCGTTAACCTGGGTTTGATCACCCT<br>GTAA
yebF	ggatcc**tttaagaaggagatatacat**ATGAAAAAAAGAGGGGCGTTTTTAGGGCTGTTGTTGGTTTCTGCC<br>TGCGCATCAGTTTTCGCTGCCAATAATGAAACCAGCAAGTCGGTCACTTTCCCAAAGTGTG<br>AAGATCTGGATGCTGCCGGAATTGCCGCGAGCGTAAAACGTGATTATCAACAAAATCGCG<br>TGGCGCGTTGGGCAGATGATCAAAAAATTGTCGGTCAGGCCGATCCCGTGGCTTGGGTCAG<br>TTTGCAGGACATTCAGGGTAAAGATGATAAATGGTCAGTACCGCTAACCGTGCGTGGTAA<br>AAGTGCCGATATTCATTACCAGGTCAGCGTGGACTGCAAAGCGGGAATGGCGGAATATCA<br>GCGGCGTCACCACCATCACCATCACTAActcgag
yebF -$(G_4S)_2$-*PA2-GNU7*	ggatcc**tttaagaaggagatatacat**ATGAAAAAAAGAGGGGCGTTTTTAGGGCTGTTGTTGGTTTCTGCC<br>TGCGCATCAGTTTTCGCTGCCAATAATGAAACCAGCAAGTCGGTCACTTTCCCAAAGTGTG<br>AAGATCTGGATGCTGCCGGAATTGCCGCGAGCGTAAAACGTGATTATCAACAAAATCGCG<br>TGGCGCGTTGGGCAGATGATCAAAAAATTGTCGGTCAGGCCGATCCCGTGGCTTGGGTCAG<br>TTTGCAGGACATTCAGGGTAAAGATGATAAATGGTCAGTACCGCTAACCGTGCGTGGTAA<br>AAGTGCCGATATTCATTACCAGGTCAGCGTGGACTGCAAAGCGGGAATGGCGGAATATCA<br>GCGGCGTGGTGGCGGTGGTAGCGGTGGCGGCGGCTCCAGCCAGCGTAAACTGGCTGCTAA<br>ACTGACCAGCAAAGGCGGTGGCCGTCTGCTGCGTCCGCTGCTGCAGCTGCTGAAACAGAA<br>ACTGCGTCACCACCATCACCATCACTAActcgag
SP_{mccV}-*PA2-GNU7*	gaattcaaaagatct**tttaagaaggagatatacat**ATGAGAACTCTGACTCTAAATGAATTAGATTCTGTTTCT<br>GGTGGTAGCCAGCGTAAACTGGCTGCTAAACTGACCAGCAAAGGCGGTGGCCGTCTGCTG<br>CGTCCGCTGCTGCAGCTGCTGAAACAGAAACTGCGTCACCACCATCACCATCACTAAggatcc<br>aaactcgag

cvaAB	gaattcaaaagatcttctcctatgttgtatgtttatatgattttccttgaaacatataatgcaaattttcgatttattttccatcattaatccagataaacaa<br>caaactaatagtatgcaaggagacattatttgtttcgccaggatgctttagaaaacagaaaaATGAAGTGGCAGGGACGGGC<br>AATATTACTTCCCGGAATACCACTGTGGTTAATCATGCTGGGAAGCATTGTGTTTATTACGG<br>CATTTCTGATGTTCATTATTGTTGGTACCTATAGCCGCCGTGTTAATGTCAGTGGTGAGGTC<br>ACAACCTGGCCAAGAGCTGTCAATATATATTCAGGTGTACAGGGATTTGTTGTCAGGCAGT<br>TTGTTCATGAAGGGCAGTTGATAAAAAAAGGGGATCCTGTTTATCTGATTGACATCAGTAA<br>AAGTACACGCAATGGTATTGTCACTGATAATCATCGCCGGGATATAGAAAACCAGCTGGTT<br>CGTGTGGACAACATTATTTCCCGTCTGGAAGAAAGTAAAAAAATAACGCTAGATACCCTG<br>GAAAAACAACGTCTGCAATACACAGATGCGTTCCGTCGCTCATCAGACATTATACAGCGT<br>GCAGAGGAAGGATAAAAATAATGAAAAATAATATGGAGAATTACAGATACTATCAGTC<br>AAAAGGACTGATTAATAAAGATCAATTAACTAACCAAGTTGCATTATATTATCAACAACA<br>AAACAACCTTCTCAGTCTGAGCGGACAAAATGAACAAAATGCCCTGCAGATAACCACTCT<br>GGAGAGTCAGATTCAGACTCAGGCAGCAGATTTTGATAATCGTATCTATCAGATGGAACTG<br>CAACGACTCGAATTGCAGAAAGAACTGGTTAACACTGATGTGGAAGGCGAAATCATTATC<br>CGGGCGTTGTCTGACGGGAAAGTTGACTCCCTGAGTGTCACTGTAGGGCAAATGGTCAATA<br>CCGGAGACAGCCTTCTGCAGGTTATTCCTGAGAACATTGAAAACTATTATCTTATTCTCTGG<br>GTCCCGAATGATGCTGTTCCTTATATTTCGGCTGGTGACAAAGTGAATATTCGTTATGAAGC<br>CTTCCCCTCAGAAAAATTTGGGCAGTTCTCTGCTACGGTTAAAACTATATCCAGGACTCCTG<br>CGTCAACACAGGAAATGTTGACCTATAAGGGAGCACCTCAAAATACGCCGGGTGCCTCTG<br>TTCCCTGGTATAAAGTCATTGCGACGCCTGAAAAGCAGATAATCAGGTATGACGAAAAAT<br>ACCTCCCTCTGGAAAATGGAATGAAAGCCGAAAGTACACTATTTCTGGAAAAAAGGCGTA<br>TTTACCAGTGGATGCTTTCTCCTTTCTATGACATGAAACACAGTGCAACAGGACCGATCAA<br>TGACTAACAGGAATTTCAGACAAATTATAAATCTGCTTGATTTGCGCTGGCAACGTCGTGTT<br>CCGGTTATTCATCAGACGGAGACCGCTGAATGTGGACTGGCCTGCCTAGCAATGATATGCG<br>GTCATTTTGGTAAGAATATTGACCTGATATATCTTCGCCGGAAGTTTAATCTCTCTGCCCGT<br>GGAGCAACCCTTGCAGGAATCAATGGAATAGCGGAGCAACTGGGGATGGCCACCCGGGC<br>TCTTTCACTGGAGTTGGATGAACTTCGAGTCCTCAAAACGCCGTGTATTCTCCACTGGGATT<br>TCAGTCACTTCGTCGTTCTGGTCAGCGTAAAGCGTAACCGTTATGTACTGCATGATCCGGCC<br>AGGGGCATAAGATATATCAGCCGGGAGGAAATGAGCCGATATTTTACAGGCGTTGCACTT<br>GAGGTCTGGCCCGGAAGTGAATTCCAGTCGGAAACCCTGCAGACCCGCATAAGTCTTCGTT<br>CACTGATTAACAGTATTTACGGTATTAAAAGAACGCTGGCGAAAATTTTCTGTCTGTCAGTT<br>GTAATTGAAGCAATCAATCTGCTAATGCCGGTGGGGACACAGCTGGTTATGGATCATGCTA<br>TTCCTGCGGGGGACAGAGGGCTACTGACGCTAATTTCTGCTGCTCTTATGTTTTTTATATTAC<br>TCAAAGCTGCAACGAGTACGCTGCGCGCATGGTCTTCACTGGTTATGAGCACGCTCATCAA<br>TGTACAGTGGCAGTCGGGGCTGTTCGATCATCTTCTCAGACTACCGCTGGCGTTTTTTGAAC<br>GCCGAAAATTAGGTGATATCCAGTCACGTTTTGACTCCCTTGACACATTGAGGGCCACATT<br>TACCACCAGTGTGATCGGGTTCATAATGGACAGCATTATGGTTGTCGGTGTTTGTGTGATGA<br>TGCTGTTATACGGAGGATATCTCACCTGGATAGTTCTCTGCTTTACCACAATTTACATTTTTA<br>TTCGACTGGTGACATACGGCAATTACCGACAGATATCAGAAGAATGTCTTGTCAGGGAGG<br>CCCGTGCCGCCTCCTATTTTATGGAAACATTATATGGTATTGCCACGGTAAAAATCCAGGG<br>GATGGTCGGAATTCGGGGGGCACACTGGCTTAATATGAAAATAGATGCGATAAATTCGGG<br>TATTAAGCTAACCAGGATGGATTTGCTCTTCGGAGGAATAAATACCTTTGTTACCGCCTGTG<br>ATCAGATTGTAATTTTATGGCTGGGAGCAGGCCTTGTGATCGATAATCAGATGACAATAGG<br>AATGTTTGTAGCGTTTAGTTCTTTTCGTGGGCAGTTTTCGGAAAGAGTTGCCTCTCTGACCA<br>GTTTTCTTCTTCAGCTAAGAATAATGAGTCTGCACAATGAGCGCATTGCAGATATTGCATTA<br>CATGAAAAGGAGGAAAAGAAACCTGAAATTGAAATCGTTGCTGATATGGGGCCAATATCC<br>CTGGAAACCAATGGTTTAAGCTATCGTTATGACAGTCAGTCAGCACCGATATTCAGTGCTC<br>TGAGTTTATCTGTAGCTCCGGGGGAAAGTGTGGCTATAACTGGTGCTTCCGGTGCGGGAAA<br>AACCACATTAATGAAAGTACTATGTGGACTATTTGAACCTGATAGCGGGAGGGTACTGATA<br>AATGGTATAGATATACGCCAAATTGGAATAAATAATTATCACCGGATGATAGCCTGTGTTA<br>TGCAGGATGACCGGCTATTTTCAGGCTCAATTCGTGAAAATATCTGTGGTTTTGCAGAGGA

	AATGGATGAAGAGTGGATGGTAGAATGTGCCAGAGCAAGTCATATTCATGATGTTATAAT<br>GAATATGCCAATGGGATATGAAACATTAATAGGTGAACTTGGGGAAGGTCTTTCTGGCGGT<br>CAAAAACAGCGTATATTTATTGCACGAGCCTTATACCGGAAACCAGGAATATTATTTATGG<br>ATGAGGCAACCAGTGCTCTTGATTCAGAGAGTGAACATTTCGTGAATGTTGCCATAAAAAA<br>CATGAATATCACCAGGGTAATTATTGCACACAGAGAAACAACGTTGAGAACTGTTGATAG<br>AGTTATTTCTATTTAAaccatagaggaattacaagcgtatgaggaatatttcttcctgttataattcctcgttatgctcagatatctgttg<br>gaggtggaatggaagatagacaatccaccaagaagaaatatcattctgtgtggattgtccaataactgttctttcggatccaaactcgag

* Gene coding sequences are shown in capital letters. RBS sequences are in bold, and restriction enzyme sites are underlined
